# Easing the transition into pathology residency: four years of experience implementing and optimizing an integrated anatomic pathology onboarding course tied to core ACGME competencies

**DOI:** 10.1016/j.acpath.2025.100231

**Published:** 2025-12-28

**Authors:** Dijana Poljak, Bronwyn Bryant, Bei Zhang, Agnes Balla

**Affiliations:** a Department of Pathology and Laboratory Medicine, The University of Vermont Medical Center, Burlington, VT, USA; b Department of Pathology and Laboratory Medicine, University of Rochester, Rochester, NY, USA

**Keywords:** Anatomic pathology, Boot camp, Onboarding, Transition to residency

## Abstract

Exposure to the field of Anatomic Pathology continues to be minimal during the medical school years. With the increasing depth of medical knowledge and the diversity of tests performed in the laboratory, incoming residents are often underprepared to fulfill the common clinical and professional responsibilities required by residency. Initially, a course with 16 sessions and later expanded to 24 sessions was designed for incoming pathology trainees spanning from July to August (2021–2024). After feedback from the first year, several sessions were modified and improved including expansion of content on how to dictate reports, populate College of American Pathologists (CAP) cancer synoptic reports, and how to build and use quick texts to increase efficiency. In the most recent iteration of the course, the new Histology Primer series developed by the American Board of Pathology was incorporated. To make this course sustainable in future years and to encourage peer teaching, senior residents were involved in teaching the expanded course content in the second year of this series. Learners completed pre- and post-course objective assessments and a separate course evaluation. The course content was aligned to address 20 of 21 milestones (Version 2) within all 6 Accreditation Council for Graduate Medical Education (ACGME) competencies.

## Introduction

The field of Anatomic Pathology has undergone significant transformation in the last decade. Advances in immunohistochemistry, molecular diagnostic tools and bioinformatics have transformed a field that was largely dependent on the exam of microscopic slides to one that relies on an ever-growing number of complex ancillary tests to reach an integrated diagnosis. These complex integrated diagnoses not only have critical impact on patient management algorithms but also drive the development of new drugs and clinical trials, which serves as the backbone of precision medicine. As in other fields of medicine, the exponential increase in knowledge that has become available with technological advances has led to subspecialization in the field. Trainees are being asked to learn more information without an increase in time-in-training, while faced with the pressure of becoming good morphologists and general surgical pathologists. These combined pressures on new trainees often overwhelm and cause a lack of understanding of how the pieces of the puzzle fit together and what to prioritize to become a competent practicing pathologist.

Pathology residency is different from every other medical specialty residency by virtue of the type of work that is performed and the content that needs to be learned. With the evolution of integrated medical school curricula, most medical schools incorporate pathology in the preclinical years by organ system. Many trainees start residency without having studied histology for several years, if at all.^1^^,^^2^ The content and amount of time devoted to teaching histology varies widely across medical schools.^1^^,^^3^ Clinical electives in medical school rarely involve rotations through the pathology laboratory. For all these reasons, medical students entering pathology residency rarely have similar experiences and may not have accrued the basic knowledge necessary to perform their duties on their residency rotations. Once trainees embark on training, they are often assigned to diverse rotations in the laboratory where expectations are not always clearly communicated leading to perceived lapses in professionalism or falling short in meeting level 1 milestones. Due to the disjointed nature of training, it can take longer for a resident to reach level 1 on certain milestones if they are not explicitly taught or primed to reflect on this early on in training.

Finally, while the importance of the healthcare team on hospital floors is emphasized repeatedly in medical school, teamwork in the pathology laboratory is rarely a point of emphasis in training. On the hospital floors, students quickly learn the importance of communication and coordination of care across an interdisciplinary team. While this same dependence on teamwork exists in the laboratory, the pathology healthcare teams have vastly different roles and areas of expertise. Incoming pathology residents have rarely been exposed to their teammates in the laboratory, which include clinical laboratory scientists, histotechnologists, cytologists (cytotechnologists), pathologists’ assistants, laboratory managers, and lab administrative staff. This lack of familiarity with key personnel can lead to lapses in communication and lack of understanding of how to integrate into the pathology team.

As such, with the purpose of orienting all new trainees equally to these new roles and residency experiences, we describe a four-year experience building an onboarding anatomic pathology curriculum for new pathology trainees and post-sophomore student fellows in pathology centered on teaching to key level 1 ACGME milestones within all 6 competencies. The assessment of learning and the course itself was aligned with all six core ACGME competencies including milestones on patient care, medical knowledge, systems-based practice, practice-based learning and improvement, professionalism and interpersonal and communication skills.

## Materials and methods

### Course design

Initially, a course with 16 one-hour lectures/sessions and in the subsequent years, a course with 24 one-hour lectures/sessions was designed for all incoming new pathology trainees (4 PGY-1 residents and 1–2 post-sophomore student fellows in pathology annually) spanning from July to August 2021 (first year course offered) to 2024 (fourth year course offered). Sessions occurred two to three times a week, either in person or on a virtual platform during our program’s usual noon didactic hour. The aim of each session was to focus teaching efforts on multiple ACGME milestones within at least 2 competencies, with one competency being professionalism. Lecturers were asked to address how professional behavior manifests on their service, how to communicate need for help, and reflect on what ethical principles might arise (Professionalism Milestones 1, 2 and 3). Topics included introductions to autopsy administration, the post-mortem exam, surgical pathology topics including grossing and intraoperative frozen section evaluation, neuropathology, and cytopathology. Each of these included a presentation that introduced the service, its workflow and team members, the resident responsibilities, and performance expectations (an example outline of the “Surgical Pathology Grossing Basics” session is provided as Supplementary Material 1). In addition to the professionalism competency, each of these sessions invoked discussion of multiple level 1 milestones including patient care (PC1-Reporting, PC-2 Grossing, PC4- Interpretation and Diagnosis), medical knowledge (MK2- Clinical Reasoning), systems-based practice (SBP-1 Patient Safety and Quality Improvement, SBP-2 Systems Navigation for Patient Centered Care), practice-based learning and improvement (PBLI-2 Reflective Practice and Commitment to Personal Growth) and interpersonal and communication skills (ICS3- Communication with Health Care Systems). Additional topics included evaluations in residency, useful online resources and books, how to engage in research, and giving/receiving feedback (Table 1). The topics of each session were chosen to align with the Anatomic Pathology services that our residents would be rotating through during their first year. The sessions that were not specifically service-oriented (research, feedback, resources, etc.) were chosen as supplemental topics that were not otherwise covered within the didactic curriculum or department orientation but were deemed necessary by program leadership and previous resident experiences. Depending on the topic, these sessions were taught by faculty, pathologists' assistants, or senior residents. The PGY2-4 residents participate in a board review style didactic curriculum for the summer, allowing for these sessions to be given solely for new trainees.

**Table 1:** Each session is mapped to the six general ACGME Competencies.

	Topic	Instructor	ACGME Competency	ACGME Milestones
**Week 1**	Introduction to Surgical Pathology Service	Faculty	PC, MK, SBP, PBLI, P, ICS	**PC4** Interpretation and Diagnosis **MK2** Clinical Reasoning **SBP1** Patient Safety and Quality Improvement **SBP2** Systems Navigation for Patient-Centered Care **SBP3** Physician Role in Health Care System **PBLI2** Reflective Practice and Commitment to Personal Growth **P1** Professional Behavior and Ethical Principles **P2** Accountability and Conscientiousness **P3** Self-Awareness and Help-Seeking **ICS3** Communication within Health Care Systems
	Surgical Pathology Grossing Basics	Faculty	PC, MK, SBP, PBLI, P, ICS	**PC1** Reporting **PC2** Grossing **PC4** Interpretation and Diagnosis **MK2** Clinical Reasoning **SBP1** Patient Safety and Quality Improvement **SBP2** Systems Navigation and Patient-Centered Care **PBLI2** Reflective Practice and Commitment to Personal Growth **P3** Self-Awareness and Help-Seeking **ICS3** Communication within Healthcare Systems
	Basic histology session: Upper/Lower Gastrointestinal tract	Resident	MK, PC	**MK1** Diagnostic Knowledge **PC4** Interpretation and Diagnosis
**Week 2**	Tips and Tricks for Dictating Cases	Resident	PC	**PC1** Reporting
	Adult Autopsy Workflow and Administration	Resident	PC, SBP, P, ICS	**PC1** Reporting **PC3** Clinical Consultation including On-Call Interactions **PC6** Autopsy **SBP2** Systems Navigation for Patient-Centered Care **SBP3** Physician Role in Health Care System **P1** Professional Behavior and Ethical Principles **ICS1** Patient and Family-Centered Communication **ICS3** Communication within Health Care Systems
	Basic Histology Session: Gynecology	Resident	MK, PC	**MK1** Diagnostic Knowledge **PC4** Interpretation and Diagnosis
**Week 3**	Basics of Performing an Adult Autopsy	Pathologists’ Assistant	PC, MK, P	**PC2** Grossing **PC4** Interpretation and Diagnosis **PC6** Autopsy **MK1** Diagnostic Knowledge **MK2** Clinical Reasoning **P1** Professional Behavior and Ethical Principles
	Approach to Pediatric and Fetal Autopsy	Faculty	PC, SBP, MK, P, ICS	**PC2** Grossing **PC4** Interpretation and Diagnosis **PC6** Autopsy **MK1** Diagnostic Knowledge **MK2** Clinical Reasoning **SBP2** Systems Navigation for Patient-Centered Care, **SBP3** Physician Role in Health Care System **P1** Professional Behavior and Ethical Principle **ICS1** Patient and Family-Centered Communication **ICS3** Communication within Health Care Systems
	Basic Histology Session: Placenta	Resident	MK, PC	**MK1** Diagnostic Knowledge **PC4** Interpretation and Diagnosis
**Week 4**	Introduction to Exam of Neuropathology Specimens	Faculty	PC, MK, P	**PC1** Reporting **PC2** Grossing **PC4** Interpretation and Diagnosis **MK1** Diagnostic Knowledge **P2** Accountability and Conscientiousness
	Introduction to Intraoperative Tissue Handling	Faculty	PC, MK, SBP, P, ICS	**PC1** Reporting **PC2** Grossing **PC3** Clinical Consultation, including On-Call Interactions **PC4** Interpretation and Diagnosis **PC5** Intra-operative consultation, including frozen section **MK2** Clinical Reasoning **SBP3** Physician Role in Health Care System **P3** Self-Awareness and Help-Seeking **ICS2** Interprofessional and Team Communication **ICS3** Communication within Health Care Systems
	Basic Histology Session: Solid Organ Gastrointestinal System	Resident	MK, PC	**MK1** Diagnostic Knowledge **PC4** Interpretation and Diagnosis
**Week 5**	Evaluation in Residency (New Innovations, RISE exam), Uknowns conferences	Faculty/Resident	ICS	**ICS2** Interprofessional and Team Communication **P3** Self-awareness and Help-Seeking
	Intro to Multidisciplinary Conferences and Anatomic Pathology Resources	Resident	SBP, ICS	**SBP1** Patient Safety and Quality Improvement **SBP2** Systems navigation for Patient-Centered Care **SBP3** Physician Role in Health Care System **ICS1** Patient and Family Centered Communication **ICS2** Interprofessional and Team Communication **ICS3** Communication within Heath Care Systems
	Basic Histology Session: Genitourinary	Resident	MK, PC	**MK1** Diagnostic Knowledge **PC4** Interpretation and Diagnosis
**Week 6**	Quality Assessment (QA) in Anatomic Pathology	Faculty	SBP	**SBP1** Patient Safety and Quality Improvement **SBP5** Accreditation, Compliance and Quality
	How to Be an Academic Pathologist- Research in Residency	Faculty	PBL, P	**PBL1** Evidence-based Practice and Scholarship **P1** Professional Behavior and Ethical Principles
	Basic Histology Session: Pulmonary/Cardiac	Resident	MK, PC	**MK1** Diagnostic Knowledge **PC4** Interpretation and Diagnosis
**Week 7**	Anatomic Pathology Curriculum, Timeline	Faculty/Resident	P	**P3** Self-awareness and Help seeking
	Evaluations: Giving and Receiving Feedback	Faculty	ICS, P	**ICS2** Interprofessional and Team Communication **P3** Self-awareness and Help-Seeking
	Basic Histology Session: Breast	Resident	MK, PC	**MK1** Diagnostic Knowledge **PC4** Interpretation and Diagnosis
**Week 8**	Introduction to the Dermatopathology Service	Faculty	PC, MK, SBP, P, ICS	**PC1** Reporting **PC3** Clinical Consultation, including On-call interactions **PC4** Interpretation and Diagnosis, **PC5** Intra-Operative Consultation, including Frozen Section **MK2** Clinical Reasoning, **SBP3** Role in Health Care System **P1** Behavior and Ethical Principles **ICS2** Interprofessional and Team Communication
	Introduction to Dermatopathology Specimen Grossing	Faculty	PC	**PC1** Reporting **PC2** Grossing
	Basic Histology Session: Dermatopathology	Resident	MK, PC	**MK1** Diagnostic Knowledge **PC4** Interpretation and Diagnosis

ICS, Interpersonal and Communication Skills; MK, Medical Knowledge; SBP, Systems-Based Practice, PBLI, Practice Based Learning and Improvement; PC, Patient Care; SBP, Systems-Based Practice.

Following feedback from the first year, several sessions were modified or added, namely the addition of eight one-hour basic histology sessions reviewing major organ systems was incorporated. These basic histology sessions were taught by senior residents using physical slides or the Larner College of Medicine Virtual Microscope slides (option was given to the resident leading the session to choose their preferred format). Summative handouts were created by a senior resident on each organ system and provided for each system. For the first two years of the histology series, learners were provided the summative handouts as prereading and expected to come prepared to participate at an interactive slide session using the virtual microscope or physical slides. Upon introduction of the ABPath Histology Primer^4^ modules, the participants were expected to complete each module prior to the interactive session for each preselected topic. Simultaneously, at the beginning of the course, senior residents were recruited to sign up for each session, with a goal to achieve a 1:1 or 1:2 teacher: learner ratio, to allow for more individualized focus for each learner. The senior residents were also encouraged to complete the modules to ensure they themselves could cover the necessary topics and simultaneously fill any gaps in their own knowledge. Following each session, learners were expected to complete the post-module quiz on the ABPath Histology Primer website and forward the results to the program director.

A tips and tricks for dictating cases session was also added in the second year the course was offered. The session was held by two senior residents and was a hands-on interactive experience demonstrating how to input diagnoses in the electronic medical record (EMR) (Epic at our institution), run reports to obtain sign out examples (with step-by-step references provided for future reference), and how to access common “SmartPhrases” (i.e. commonly used verbiage for the most common diagnoses). The session allowed participants to practice on test cases using the SmartPhrases, as well as the dictation software (M∗Modal at our institution). Participants were also provided with a reference card containing the most commonly used phrases, references, and framework for a surgical pathology report (an example of this is provided as Supplementary Material 2).

### Course objectives

1. Model behaviors that support the culture of the University of Vermont Medical Center Pathology program: respect, professionalism, effective communication, transparency, and truthfulness (ACGME Competency: professionalism, interpersonal and communication skills)
2. Describe the resident role in the major core Anatomic Pathology (AP) rotations and explain the expectations for successful completion of each rotation. (ACGME competency: patient care, systems-based practice, practice-based learning and improvement)
3. Demonstrate fundamental knowledge in each of the above core rotations, which will assist in minimizing errors and maximizing delivery of high-quality care. (ACGME competency: medical knowledge)
4. Review basic histology for major organ systems to optimize success when starting surgical pathology rotations. (ACGME competency: medical knowledge)

### Course assessments

An objective assessment consisting of the same 10 questions was administered at the beginning of the course and within one month of completion of the course. The intent of the questions was to assess baseline knowledge and determine whether the trainees retained vital basic concepts presented from each core AP rotation lecture (surgical pathology, neuropathology, cytopathology, autopsy, and dermatopathology). The written assessment was designed to be detail-oriented and relevant to clinical practice but was not intended to be comprehensive and inclusive of content from each session. Its purpose was to serve as a metric for evaluating the retention of fundamental medical knowledge over this time period.

### Course evaluation

For continued improvement, a course evaluation assessing quality using a Likert scale was designed to assess the quality of the content for each session. Additionally, perceived utility of each session in meeting the outlined objectives was also similarly assessed using a Likert scale. The evaluation concluded with an open-ended question soliciting feedback for improvement of the course.

## Results

**Fig. 1:**
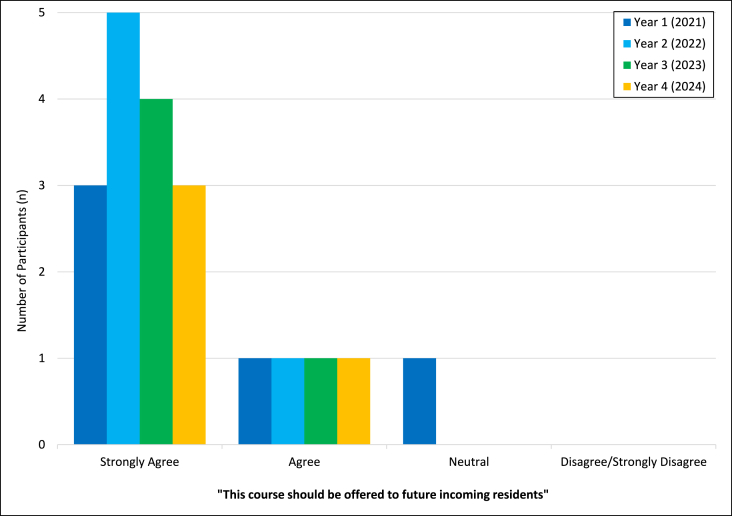
Number of participants agreeing with continuation of the course for future pathology residents (n = 20).

The average score of all participants (n = 20) was 95 % on the objective assessment following completion of the course (range of 50–100 %). Average score obtained on the assessment at the start of the course (in the third and fourth year that it was offered) was 79 % (with a range of 50–100 %, n = 10), demonstrating improvement from pre and post assessments. The most missed questions were related to grossing of dermatopathology specimens and differences between pediatric and adult autopsies. Nearly all participants agreed or strongly agreed that the course should be offered to future incoming first years (Fig. 1), with the only exception being one neutral assessment in 2021 (first year course was offered).

**Fig. 2:**
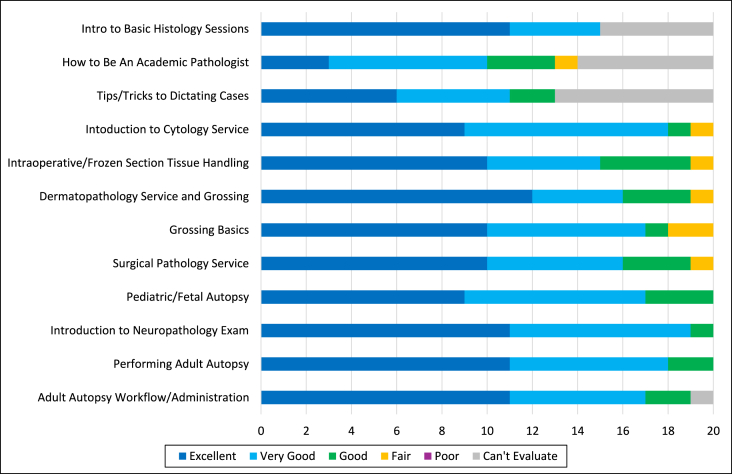
Participant rating of the quality of the content presented for each of the sessions collated over four years (n = 20).

Overall, when asked about the quality of the content presented using a 5-point Likert scale, 85 % of the ratings were reported as excellent or very good (Fig. 2). Sessions with the most favorable ratings (very good to excellent) included introduction to dermatopathology service and grossing, introduction to the neuropathology exam, performing an adult autopsy, and adult autopsy workflow and administration. None of the sessions received a Poor rating.

**Fig. 3:**
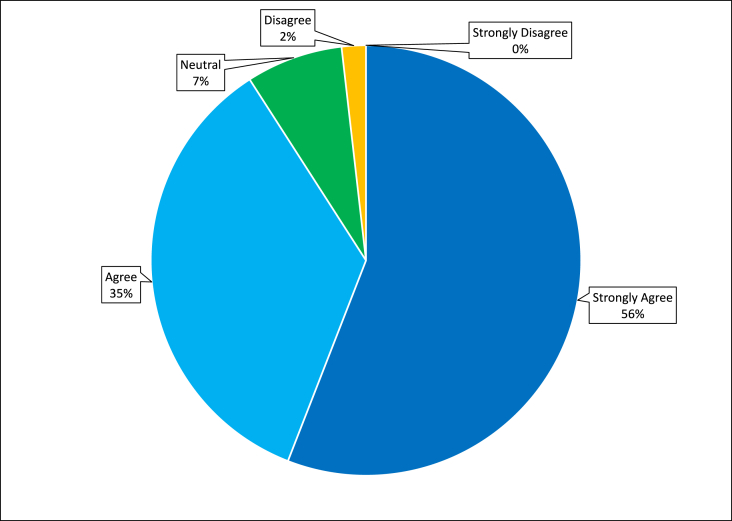
Assessment of the summative overall perceived positive utility of all sessions combined over four years (n = 20).

**Fig. 4:**
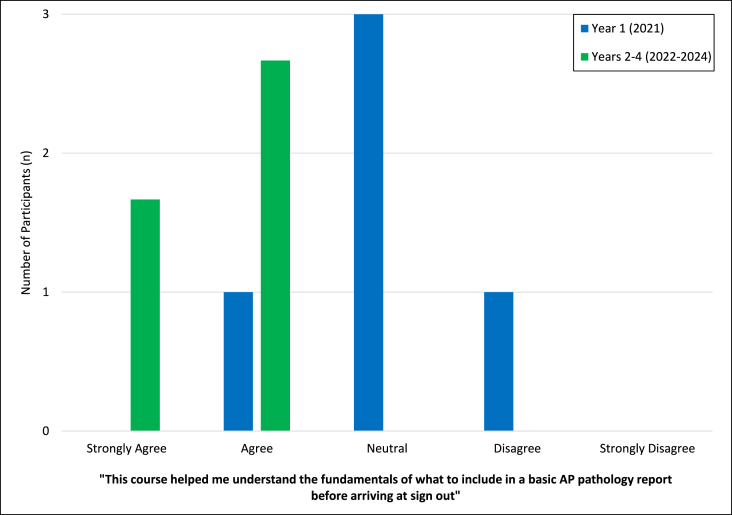
Comparison of year 1 (2021) versus years 2–4 (2022–2024) (averaged) of participants who agreed that the course helped them understand the fundamentals of what to include in a basic Anatomic Pathology (AP) pathology report.

Assessment of the perceived utility of each session demonstrated overall favorable responses, with 91 % percent of learners responding with agree or strongly agree (Fig. 3). Participants in all years had positive reviews regarding sessions on grossing, feedback, and the anatomic pathology team. Notably, all participants agreed or strongly agreed with the statements “this course highlighted how grossing impacts the ability of a pathologist to make an accurate diagnosis and support the highest quality of patient care” and “this course enhanced my understanding of how I fit into the anatomic pathology team which includes clinicians, attending pathologist, technologists, pathologists’ assistants and support staff” (ACGME Milestones Patient Care 1, 2, 4, Systems-Based Practice 2). All participants agreed or strongly agreed that the added intro to basic histology sessions were a useful review of normal and benign histology (n = 15). After the first-year evaluations revealed that only 20 % of participants agreed that they felt comfortable with the fundamentals of an AP report, an additional session on tips and tricks of dictating cases was added in year two. This resulted in all participants in the subsequent years (years 2–4 that the course was offered) stating that they strongly agreed or agreed that the course helped them understand the fundamentals of an AP report for optimal patient care (meeting ACGME milestone Patient Care 1: Reporting) (Fig. 4). All other sessions had similar ratings from year to year (data not shown).

## Discussion

Publications on onboarding or “bootcamp” experiences for pathology trainees are limited. The few published reports on institutional experiences describe a combination of online modules, lectures, and grossing/histology review.^5^, ^6^, ^7^ These generally describe an experience lasting from weeks to up to 3 months, whereby residents are immersed in the bootcamp experience without other clinical responsibilities. Similar to our curriculum, a few studies describe intercalated lectures set aside for PGY-1s only. However, to the best of our knowledge, our curriculum is the first of its kind to span the first two months of residency while residents embark on clinical duties and concurrently focus on key aspects of the ACGME milestones. Furthermore, we demonstrate that onboarding courses are not static and rigorous annual evaluations and feedback from the residents is necessary for continual optimization. As innovative technologies and shifts in practice emerge, we must adapt our teaching to reflect these changes. Evaluations by participants also commented that doing this course as a cohort of first-year trainees also fostered collegiality and relationships amongst one another as first year residents, which served to support help seeking (Milestone; Professionalism 3 Self-awareness and help-seeking).

We designed this course using the ACGME Milestones and competencies as our blueprint recognizing that many of the Level 1 competencies could be achieved by trainees by specifically teaching to these topics and priming them early on in training. For example, the introduction to grossing session was designed to target several competencies within several milestones including Patient Care, Practice-based Learning and Improvement, Systems-based Practice, and Professionalism. This session discussed the impact of grossing on pathologic staging and the impact of margin orientation (*en face*, reverse *en face,* or perpendicular) on reporting and subsequent treatment decisions. College of American Pathologists (CAP) synoptic reporting was reviewed and residents learned that most of the elements in these reports are derived from the gross exam and from subsequently submitted histology slides. Internal grossing protocols, grossing manuals, and other resources were reviewed that provide fundamentals of grossing. Trainees were primed on the importance of avoiding sample misidentification by providing an example of a real-life patient safety event. The importance of accountability, conscientious behavior, and ethical principles were discussed in this context. The importance of time management and planning during times of high volume were addressed and trainees were empowered to reach out to faculty on service or peers if their grossing volume was excessive to provide quality patient care. The importance of integrating radiologic and clinical findings with a gross exam was emphasized and trainees were coached on the need to be able to explain the clinical question that needs to be answered with each case before starting grossing. Appropriate resources available in the electronic health record (Epic) to answer these questions (operative notes, radiology, labs, etc.) were reviewed. While it is not possible to review in depth which milestones and competencies were addressed in each session, this session is an example of how trainees can demonstrate meeting level 1 competencies through various anatomic pathology services and activities when made aware of them (grossing, intraoperative evaluation, autopsy, cytopathology and on site evaluations, etc.). Table 1 lists which competencies and milestones were addressed in each session.

We concluded that one of the most valuable aspects of this curriculum is the involvement of senior residents in teaching and their role in the histology sessions, a method previously established to be an effective strategy for successful transition to residency curricula.^8^ Studies have demonstrated that medical students only retain about 50 % of their basic science/histology knowledge.^9^ Paired with the knowledge that there has been a steady decline in histology teaching within medical school curricula,^3^ this highlighted the need to include histology teaching with our curriculum and build on the medical knowledge competency. By having senior residents present this material—who are closer to the basic content than most attendings—the content is much more aligned to PGY-1 level learning. Rather than just watching videos passively, new residents can engage with the material prior to each session by using the provided prereading material and/or the ABPath Histology Primer modules.^4^ They come prepared to each session and ask questions, and as such, the sessions are much more interactive. These sessions have routinely been the highest rated. Additionally, junior residents are inspired by their peers and look up to them as role models.^10^^,^^11^ Senior resident engagement also ensures future continuation of the course and allows attending pathologists to focus their teaching on higher level topics. Lastly, but equally important, senior residents benefit greatly from practicing teaching at the microscope. This is a skill that takes practice and is of great utility for those seeking a career in academic medicine where slide teaching sessions and presentations at tumor boards and national meetings are an integral part of the job. Previous studies have also demonstrated this model can sustainably benefit upper-level residents while simultaneously improving Resident In-Service Examination (RISE) scores for first year residents.^12^ Previous ACGME surveys also demonstrated that programs that included senior residents in orientation proved to be the most helpful learning method.^13^

The limitations in our study are twofold. First, we have a small sample size (an average of 4–5 new trainees per year). This sample size is a challenge for any new educational intervention for PGY-1 residents as most programs have a small number of new trainees. Another limitation is an inability to objectively assess how this intervention has broadly impacted the workflow for residents, faculty, and staff in anatomic pathology. Although our faculty have welcomed this course and expressed they feel it is important, future considerations can include assessment of faculty and staff on their perceived performance of incoming new trainees rotating onto their services. This assessment would be limited to only post-implementation performance as we do not have any way of comparing pre-implementation performance in a standardized way.

Additional limitations include the timeliness of delivery compared to when the learner is first exposed to the rotation and the overwhelmingly predominant concern also voiced by learners in the course evaluation. In our combined AP/CP residency program, it may take months before a resident rotates onto an AP service to which they have been oriented through this course, while on the other hand, some have already rotated through a service covered during the course. We have attempted to at least partly address this issue by posting all of the presented resources on a shared drive for later review.

## Conclusions

Objective and subjective evaluations showed that this course has value in providing fundamental knowledge and resources for new trainees while meeting basic competencies in several of the basic ACGME Milestones early in training. Nearly all learners (95 %) agreed that this course should be continued in future years. An AP onboarding course can ease the transition to residency while providing an opportunity to establish a culture of professionalism, teamwork, and collegiality. By incorporating senior residents teaching many sessions, we can ensure sustainability of our course, as junior residents can expect to teach their peers later in training. Course evaluations can help identify gaps specific to the needs of each individual topic area. Three one-hour sessions per week integrated into the normal clinical workflow of any program makes this curriculum easy to implement at many institutions. A dedicated faculty course director and senior resident assistant course director enables continual improvement and organization of the course.

## Funding

The article processing fee for this article was funded by an Open Access Award given by the Society of ‘67, which supports the mission of the Association for Academic Pathology to produce the next generation of outstanding investigators and educational scholars in the field of pathology. This award helps to promote the publication of high-quality original scholarship in *Academic Pathology* by authors at an early stage of academic development.

## Declaration of competing interests

The authors declare that they have no known competing financial interests or personal relationships that could have appeared to influence the work reported in this paper.
