# Supplementary material for: Easing the transition into pathology residency: four years of experience implementing and optimizing an integrated anatomic pathology onboarding course tied to core ACGME competencies
Source: Acad Pathol. 2025 Dec 28;13(1):100231. doi: 10.1016/j.acpath.2025.100231 (PMC12803838; doi:10.1016/j.acpath.2025.100231)
Supplement: Multimedia component 1 [file mmc1.pdf]

Supplemental Material 1: Surgical Pathology Grossing Basics Lecture Content Outline.

1. **Introduction**
   a. Review the key steps and care coordination involved in specimen processing, including specimen collection and storage conditions in hospital and ambulatory care settings, cold ischemic time, specimen receiving, accessioning, billing, gross examination, histology, resident preview, final pathologist review and sign-out, and specimen storage/discard. (SBP2)
2. **Clinical Correlation and Specimen handling**
   a. Retrieve and interpret relevant radiologic and clinical test results from the electronic health record prior to handling cancer excision specimens. Correlate specific imaging findings (e.g., mammography, MRI, ultrasound) with gross examination features to identify key abnormalities and develop a focused differential diagnosis. (PC4, MK2, SBP2)
   b. Identify specimen types requiring special non-formalin fixatives and select the appropriate fixative based on clinical context (e.g., medical renal biopsies, lymphoma evaluation). Execute proper handling and documentation to preserve diagnostic integrity. (PC1, SBP1, MK1)
3. **How Grossing Impacts Reporting and Clinical Care**
   a. Emphasize the need to recognize and describe the normal gross appearance of specimens in order to identify deviations that warrant targeted sampling. (PC2, PC4)
   b. Explain the importance of performing accurate gross measurements, descriptions, and sampling of tissue, and explain how these practices impact downstream diagnostic reporting (PC4)
   c. Emphasize practices that maintain specimen integrity, including proper handling, and encourage consultation of grossing protocols and reference materials. (PC2
   d. Review an example of a near-miss event involving tissue misidentification. The resolution process and system safeguards designed to prevent similar errors are outlined. (SBP1)
4. **The Gross Description**
   a. Review the essential components and structure of a high-quality gross description, emphasizing clarity, completeness, and relevance to diagnostic accuracy. (PC1)
   b. Discuss the role of gross descriptions in CAP cancer synoptic reporting, highlighting how detailed gross findings directly inform key synoptic elements. (PC1)
5. **Communication, Teamwork and Professionalism in the Grossing Room**
   a. Explain the educational background, training, and professional responsibilities of pathologists' assistants, and their integral role within the pathology care team. (SBP3)
   b. Acknowledge common stressors associated with workload management in the gross room, and discuss strategies for recognizing when to seek support or escalate concerns. (PBL2, P1, P3)
   c. Describe best practices for effective communication and safe handoff of complex specimens between team members to ensure diagnostic continuity. (P1, ICS3)
