# Supplementary material for: Easing the transition into pathology residency: four years of experience implementing and optimizing an integrated anatomic pathology onboarding course tied to core ACGME competencies
Source: Acad Pathol. 2025 Dec 28;13(1):100231. doi: 10.1016/j.acpath.2025.100231 (PMC12803838; doi:10.1016/j.acpath.2025.100231)
Supplement: Multimedia component 2 [file mmc2.pdf]

Supplemental Material 2: Sign Out Guide.

The goal of this hand out is to provide trainees in their early days of surgical pathology a quick reference guide in how to put together a basic surgical pathology report. It should include the following elements:

FINAL DIAGNOSIS SECTION:

SPECIMEN*, LATERALITY, LOCATION, PROCEDURE:

- **Major diagnosis**
- **Most important findings (e.g. margins, stage)**
- **See comment (if applicable)**

*The specimen should be designated based on what the clinical team/surgeons called the specimen when it was submitted

*If an unusual name or there is discrepancy within the histology/submission and no further clarification can be obtained, you can use the terminology "Submitted as "xxx" "

COMMENT SECTION:

- Elaborate on your major diagnosis, if needed. For cases where the differential is still broad, describe why the major diagnosis is the most favored or why there is still ambiguity.
- State whether case was reviewed in consensus conference or given for consultation to another pathologist/subspecialist
- State if prior biopsies/specimens were also reviewed in conjunction with this case
- Can also use this section to describe the histology (some pathologists prefer the "Microscopy" section for this, practices vary).
- List any IHC that was used to help make the diagnosis (*this is a billing requirement*), including the name of the IHC and the interpretation (again, some pathologists may prefer to place this in the "Microscopy" section as a list, while others write a paragraph incorporating the stains into the explanation of the case).

An example of one version of this handout given to our trainees:

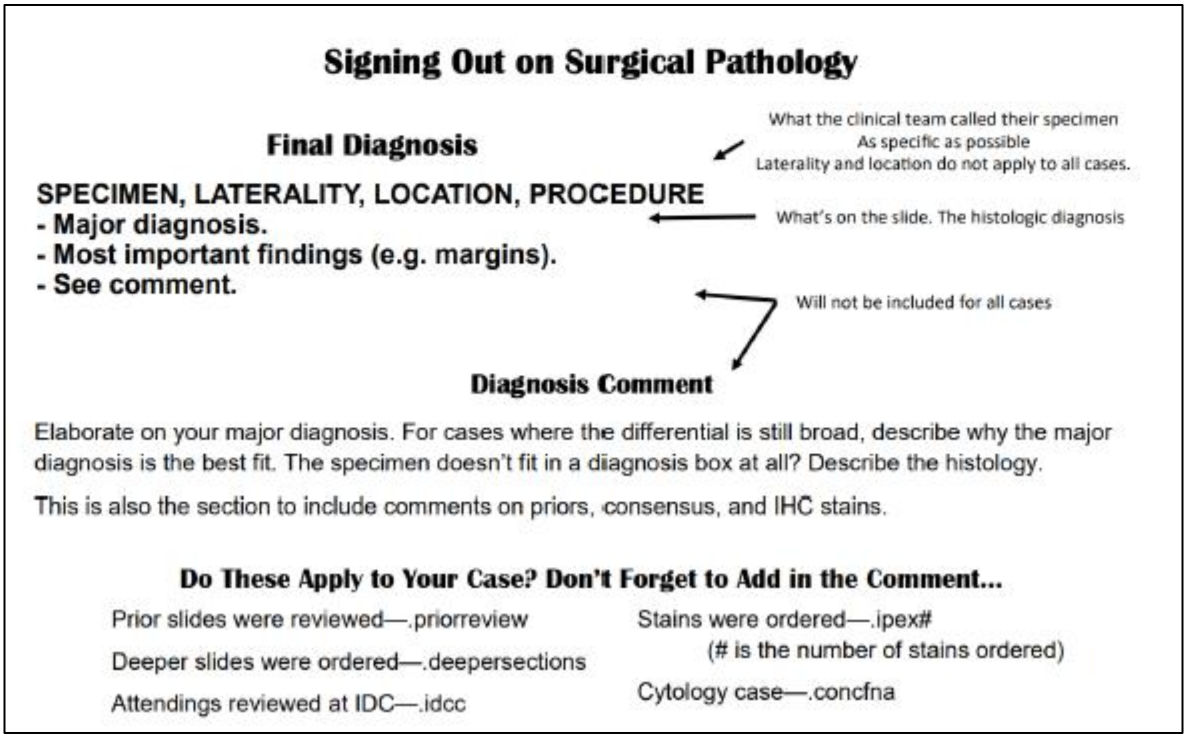

Signing Out on Surgical Pathology

Final Diagnosis

What the clinical team called their specimen
As specific as possible
Laterality and location do not apply to all cases.

SPECIMEN, LATERALITY, LOCATION, PROCEDURE
- Major diagnosis.
- Most important findings (e.g. margins).
- See comment.

What's on the slide. The histologic diagnosis

Will not be included for all cases

Diagnosis Comment

Elaborate on your major diagnosis. For cases where the differential is still broad, describe why the major diagnosis is the best fit. The specimen doesn't fit in a diagnosis box at all? Describe the histology.

This is also the section to include comments on priors, consensus, and IHC stains.

Do These Apply to Your Case? Don't Forget to Add in the Comment...

Prior slides were reviewed—.priorreview

Deeper slides were ordered—.deepersections

Attendings reviewed at IDC—.idcc

Stains were ordered—.ipex#
(# is the number of stains ordered)

Cytology case—.concfna
